# 

**Published:** 1904-06

**Authors:** 


### CONTENTS.

ORIGINAL COMMUNICATIONS—

GENITO-URINARY TUBERCULOSIS IN THE FEMALE. By E. G. Jones, M.D , Atlanta, Ga. .... 145
ANOPHELES AND MALARIA. By A. Laveran .... 153
REMOVAL OF PSOAS AND ILIAC MUSCLES OF THE LEFT SIDE FOR SARCOMA. By Geo. H. Noble, M.D., Atlanta, Ga. .... 162

EDITORIAL NOTES AND COMMENTS—

SCHOOL COMMISSIONERS STATE ASSOCIATION TO COOPERATE WITH STATE BOARD OF HEALTH .... 166
THE BARUCH-VON GYOERY DISCUSSION .... 170

NEWS AND NOTES .... 172
CONTENTS CONTINUED .... X

BOOK REVIEWS—
A System of Practical Surgery. (Von Bergman, Von Bruns, Von Mikulicz).... 176
SELECTIONS AND ABSTRACTS—
Puerperal Infection........ 177
Some Eye Complications Seen in General Diseases........ 186
Treatment of Long Bone Fractures........ 190
The Relationship of Diseases of the Bronchi and Lungs to Those of the Nose and Throat........ 194
Some Clinical Observations on the Prophilaxis Treatment of Uric Acid Conditions........ 200
The Attitude of the Medical Profession Toward Food Preservatives........ 205
Cystitis in the Female........ 207
The Specific Treatment of Tuberculosis........ 213
MEDICAL ITEMS—Continued........ xviii, xx, xxii, xxiv, xxvi, xxviii.

